# Some Thoughts on Tuberculosis

**Published:** 1910-07

**Authors:** George Brown

**Affiliations:** Atlanta, Ga.


### SOME THOUGHTS ON TUBERCULOSIS.

BY GEORGE BROWN, M. D., ATLANTA, GA.

The abstract of my paper as published in the Bulletin of the Medical Association is a little misleading. It is not my intention to write a technical paper or go into the minutiae of the incipiency of tuberculosis. I prefer to use a broader aspect of this disease, with special reference to the prevention thereof either by medical or legal means, and in order to intelligently arrive at a conclusion I would divide this paper into two parts: The first I would diagnose as the medical aspect of the prevention of tuberculosis. The second I would diagnose as the legal aspect of tuberculosis. Now, as to the medical part, I will not take up your time or tax your patience by going over this, as I have nothing startling to offer in this direction. One thing, however, I would make plain: That either the errors of physicians or the neglect of the physicians are responsible for the loss of innumerable lives. At a meeting of the Board to organize the

---

Rear before the Ga. Med. Ass'n. at Athens, Ga., April 20-22, 1910.

State Sanitarium in Atlanta last year, Dr. Jefferson Davis, of Toccoa, Ga., one of the trustees of that institution, made use of the following words. Then it was suggested that an educational feature be added to the work of this institution. Dr. Davis said, "We do not need to educate the people concerning this disease. What we need to do is to educate the doctors." No truer saying ever fell from the lips of man. The patient comes to the doctor. One of this class as soon as he diagnoses the case, if the patient is able to pay for the attention of a specialist in this line, immediately tells the patient his condition, and sends him to one. The other one holds the patient as long as he possibly can, doses him on Cod Liver Oil, quinine and various other remedies, telling the patient that he has a little "lung trouble" or a little "bronchial trouble." The patient patiently wends his way towards the cemetery at about the usual rate of speed, and towards the close of the drama the doctor gets very anxious concerning him, and he generally suggests that the patient go West; sometimes he sends him to a sanitarium nearer home. When the time comes for the patient to leave, the doctor generally informs the family "that he will be all right now, and that he will come back home as good as new." This is supposed to square the doctor with the family and the patient's friends, and the patient comes back home in the "Baggage Car Ahead." The other class of doctors have a heart to heart talk with their patient, and with the friends of the family, telling them the exact condition of the patient, and suggests that he be sent to a sanitarium for treatment. He does not fail to tell them that statistics show that from two to four per cent. of these people get well at home, and that from sixty-two to eighty-two per cent. get well in well-equipped sanitariums. If the patient is able to pay the charges, he is generally sent away in order to give him a chance for recovery, and if he is not able to pay he has to take his turn and wait for death. Now, from a plain business standpoint, I ask any man to pick out a town in the State of Georgia having two physicians and watch their career for ten years. Eventually at the end of ten years one of these physicians has the confidence and respect of the entire community, and the other one who merely diagnosed these cases as a "little lung trouble" or a

"little bronchial trouble" has either sold out and moved away, or returned again to the farm or else running a saw mill. The method of fooling these people about the disease worked all right fifteen years ago, but there is an old adage, "that the times change, and that the people change with them," and the doctor who is not able to change with the educational advancement of this disease is just as sure to lose his patients as the sun shines. Carrying the idea a little further, I wish to call attention to another element. It is the duty of every good physician to prevent this disease, and no man has gone further into the matter than an illustrious Georgian, Mr. Nathan Strauss, born at Talbotton, Ga., and the man who is responsible for all the valuable information pertaining to this milk business. It is useless to eulogize his work in regard to the pasteurization of milk. Notwithstanding the animosity and abuse which has been hurled against this man by the milk trust, he is to-day greater than any doctor that ever breathed on the American continent, and his name will be emblazoned in the Hall of Fame when his traducers shall have been forgotten, resting on the lap of Mother Earth unwept, unknown and unsung. Mr. Strauss has established at Lakewood, N. J., a Preventorium in which tubercular children, or those which show a tendency towards tuberculosis, or those who live in tubercular homes can be taken and given three or four months' treatment and sent back home in perfect health. Soon after he began this magnificent charity what was the result? A great howl went up, and an injunction was issued restraining him from opening this institution. The true history of this proceeding has never been known, as the press was never able to get it, but I will give it to you free just as the condition exists:

Nathan Strauss and Max Nathan jointly owned the Lakewood Hotel property. The Cleveland cottage, situated in a remote corner of the property was Mr. Strauss' personal property, but as transactions between Strauss and Nathan were never reduced to writing, the Cleveland cottage property was never partitioned from the hotel property by the passing of a deed. The Preventorium was established in the Cleveland Cottage by Mr. Strauss.

George J. Gould, having brought up his children, wanted to sell his great Lakewood estate, "Georgian Court," and he feared that the establishment of the Preventorium would discount the value of his property. Max Nathan, now eighty years old, is head of the pump trust that supplies hundreds of thousands of dollars worth of supplies to the Gould railroads every year. This leverage proved stronger with Max Nathan than the obligations of friendship, and of his verbal arrangement with Strauss about the Cleveland Cottage. Samuel Utermyer is counsel for the pump trust. He and George Gould induced Mr. Nathan to dispute Mr. Strauss' exclusive ownership of the Cleveland Cottage, and stirred up the Lakewood people to fight the institution. Hence the bitter war upon the Preventorium—the pump trust fighting the children at the dictation of the Gould railroad trust.

The work of Mr. Strauss has opened up a new line which is doing great good, and it shows that children who suffer from Tuberculosis or show any symptoms indicative of tuberculosis can be restored to perfect health and be made healthy citizens of the community.

Now, in regard to the legal status of the tubercular patient. These people are unable to work, and the majority of them have no means. What disposition is to be made of them? It is a question which has been satisfactorily solved in Europe, and to-day in Germany, England and France no one of these cases need go without proper medical attention. The time is fast approaching in the United States when we will be up with the times. One of the greatest tasks of the legislators is in making changes which is absolutely necessary to meet conditions existing in a new country. Is it not time to urge our legal departments that we are not only one of the greatest people in the world, but that we are one of the most progressive? All of this is true, but in many respects, I might say, in nearly all respects as far as hygiene and sanitation are concerned, we are fifty years behind the oldest countries of Europe. New settlers in a new country go through about the same cycle of improvements. They first live in log cabins, then in farm houses, then in brick, and finally in stone. As far as medical legislation is concerned, we are still in the log cabin stage. I asked the magnificent Board of Health of this state: "Do any of you realize that these people are doing their work on an appropriation of less than $20,000 per year? Do you realize that the State of Georgia appropriates two and a half millions of dollars per year for education; another million for pensions, and expends for the health of its citizens the magnificent sum of $20,000 alone?" Is it any wonder that to my mind the average member of the Legislature is a jackass? I wish to state emphatically that this is not my true opinion of the average legislator; my true opinion is not fit to publish, and I fear it would not be accepted in the society columns of any of our great dailies. I have no apology or explanation to make for expressing this opinion. Nay, the so-called legislator that is too ignorant or too careless to the suffering of humanity not to unlatch the purse strings of this great state to help the poor indigent sufferer has not my respect. I consider him an naught with the ring rubbed out. Take the National Government. There was expended in the years 1908-09 for past wars, and for preparation for war, four hundred and twenty-three millions of dollars. There was left for other business of the Government one hundred and eighty-one millions of dollars, 70 per cent. for one and 30 per cent. for the other of the income of the United States. The cost of a few battle ships wisely spent in the fight against tuberculosis in a few years would obliterate this disease practically from the masses of the Republic. The Secretary of the Peace Society of the State of New York says: "Our defences, adequate in 1898, are doubly equipped now. Why not let well enough alone, and buy something besides guns. No one can oppose adequate national defences. We do oppose actively the following of the prevailing national ambition." Mr. J. A. Tauney, chairman of the committee on appropriations, states that expenditures for this purpose for the coming fiscal year, 1909-10, will be greater than ever for this year. They have been increased rapidly and enormously year by year, and no one can predict beyond what increase this may not go. The question arises, what are we going to do about it? We must awaken the people to the fact that this country is on the verge of delirium on expenditures for war alone. To this one fact, every doctor, every humanitarian and every voter should obligate himself to aid in every way movements which tend to reduce this great amount of expense, in a more sane way, and aid in the upbuilding and betterment of mankind.

